# *Onchocerca jakutensis* Filariasis in Humans

**DOI:** 10.3201/eid1311.070017

**Published:** 2007-11

**Authors:** Martina Koehsler, Afschin Soleiman, Horst Aspöck, Herbert Auer, Julia Walochnik

**Affiliations:** * Medical University of Vienna, Vienna, Austria

**Keywords:** Onchocerca jakutensis, zoonosis, filariasis, PCR, paraffin-embedded tissue, lupus erythematosus, dispatch

## Abstract

We identified *Onchocerca jakutensis* as the causative agent of an unusual human filariasis in a patient with lupus erythematosus. To our knowledge, this is the first case of human infection with *O. jakutensis* and the first human case of zoonotic onchocercosis involving >1 worm.

Zoonotic filarial infestations occur worldwide, and in most reported cases the involved species are members of the genus *Dirofilaria*. However, zoonotic *Onchocerca* infections are rare and to date only 13 cases (originating from Europe, Russia, the United States, Canada, and Japan) have been described. In all of these cases only 1 immature worm was found, and the causative species was identified as *O*. *gutturosa*, *O*. *cervicalis*, *O*. *reticulata*, or *O*. *dewittei japonica* on the basis of morphologic and in some cases serologic parameters (*1*–*4*). *O*. *cervicalis* and *O*. *reticulata* are found in the ligaments of the neck and extremities of horses, *O*. *gutturosa* is typically found in the nuchal ligaments of cattle, and *O*. *dewittei japonica* is found in the distal parts of the limbs and adipose tissue of footpads of wild boars.

We identified the causative agent of a zoonotic *Onchocerca* infection with multiple nodules in a patient with systemic lupus erythematosus (SLE) who had been receiving hemodialysis. The parasite was identified in paraffin-embedded tissue samples by PCR and DNA sequence analysis.

## The Study

The patient was a 59-year-old woman with SLE who had developed multiple nodules on the neck and face over several years. Because of major renal insufficiency, she also had been receiving hemodialysis 3 times per week (3.5 hours) for >10 years. The first clinical differential diagnoses were cutaneous SLE, nephrogenous dermatopathy, calciphylaxis, and calcinosis. The clinical picture was obscured by secondary inflammations and ulcerations caused by self-inflicted trauma. Multiple sampling attempts by cutaneous core biopsies resulted in histologic diagnosis of unspecific, secondary inflammatory changes. Deep surgical excision of 1 subcutaneous nodule on the scalp indicated subcutaneous helminthosis (Figure). The patient was treated with ivermectin and subjected to 2 plastic surgeries for facial reconstruction, after which she recovered.

**Figure:**
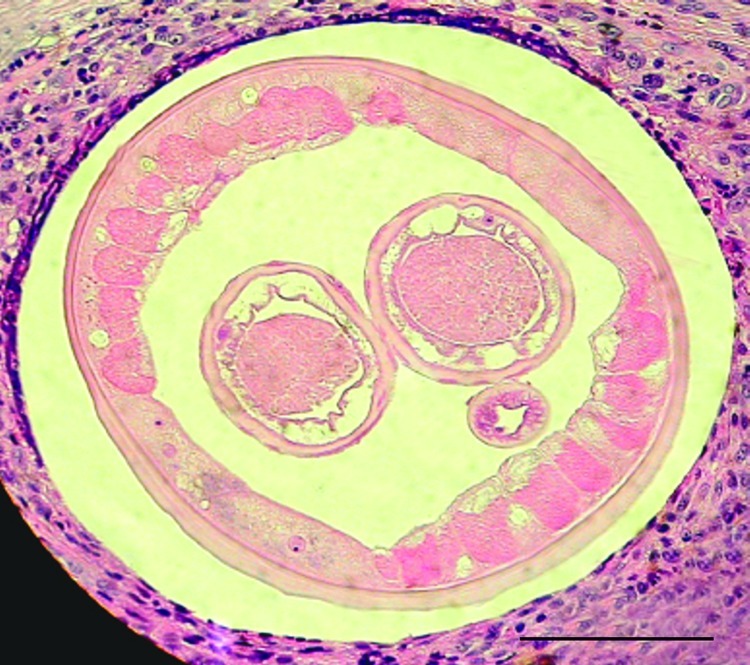
Transverse section of a female worm and surrounding tissue isolated from the patient (hematoxylin and eosin stained). Scale bar = 100 μm.

At this point, species identification of the causative agent was still pending. A history of travel anamnesis and location of the nodules indicated a possible *Dirofilaria* infection, but a specific PCR showed negative results. Morphologic features of the few available sections suggested *Onchocerca* spp. To our knowledge, multiple nodules had never been reported in cases of infection with zoonotic *Onchocerca*. Because a definitive morphologic identification of the causative nematode was not possible, molecular identification from DNA isolated from the only available material (formalin-fixed and paraffin-embedded tissue) was conducted.

To evaluate the causative genus, universal filarial primers were constructed on the basis of filarial sequences available in GenBank (primer FILfw 5′-cggtgatattggttggtctc-3′ for the first internal transcribed spacer region and primer FILrev 5′-ctagctgcgttcttcatcgatc-3′ for the 5.8S rRNA gene). PCR and sequencing were performed and a similarity matrix was calculated after multiple sequence alignment (*5*).

The DNA fragment obtained was 226 bp and showed greatest similarities to *Onchocerca* sequences, ranging from 87% to 95%. Similarities to *Wuchereria*, *Brugia*, *Mansonella*, *Dirofilaria*, and *Acanthocheilonema* were lower, ranging from 75% to 80%. Assignment to the genus *Onchocerca* was obvious, but species identification still posed a problem because published *O*. *volvulus* sequences showed higher similarities among each other (98.8%–100%) than with our sequence. The only exception was a clinical *O*. *volvulus* strain (OvNod1–3) from Bolo, Cameroon, which showed 94.8% sequence similarity. However, the authors of that report indicated that their strain might be a zoonotic *Onchocerca* sp. (*6*).

An identical thymine mononucleotide repeat motif in our strain and strain OvNod1–3, which was shorter in all *O*. *volvulus* sequences, indicated that both strains were not *O*. *volvulus* because repeat motifs have been reported to occur in species-specific patterns. The negative results with an *O*. *volvulus*–specific PCR (*6*) corroborated this assumption. Therefore, 2 additional primer pairs for *Onchocerca* spp. identification were constructed, 1 for the mitochondrial NADH dehydrogenase subunit 5 gene (OND5fw 5′-ctcctgttagttgtttggttc-3′, OND5rev 5′-gcaaacccctaccaatagc-3′) and 1 for the 16S mitochondrial rRNA gene (O16fw 5′-gcgtgatggcataaaagtagc-3′, O16rev 5′-caaccctgttaactccggag-3′), on the basis of available *Onchocerca* spp. sequences (*7*,*8*). PCR products were sequenced and similarity matrices were calculated (Tables 1, 2). The NADH amplicon was 201 bp and the 16S rRNA amplicon was 225 bp. Both amplicons unambiguously identified our strain as *O*. *jakutensis* with 100% and 99.55% sequence similarities, respectively. Sequence data were deposited in GenBank and are available under the following accession nos.: EF202184, EF202185, and EF202186.

**Table 1:** Sequence similarities in the NADH dehydrogenase subunit 5 gene in the *Onchocerca* sp. isolated in this study and other *Onchocerca* spp.*

Species	% Sequence similarity											
	*arm*	*duk*	*fas*	*fle*	*gib*	*gut*	*jak*	*lie*	*och*	*ram*	*vol*	This study
*arm*	100	94.03	91.54	91.54	92.04–92.53	86.07–92.54	92.04	91.54–92.54	89.55–93.03	81.59	93.03–94.03	92.04
*duk*		100	94.53	92.54	96.51–97.01	92.04–96.51	94.53	97.51–98.51	95.02–97.01	82.59	98.01–99.00	92.02
*fas*			100	89.05	92.04–92.53	88.56–94.03	94.53	95.02–96.02	92.04–95.52	82.59	93.53–94.53	94.53
*fle*				100	93.03–95.03	86.57–92.04	91.04	91.04–92.04	90.55–92.04	82.09	92.53–93.53	91.04
*gib*					99.5	90.05–95.02	92.04–92.54	94.03–95.52	94.03–95.52	84.08–84.58	95.52–97.01	92.04–93.54
*gut*						90.55–97.51	89.05–94.53	91.54–96.02	91.04–95.52	79.10–82.59	91.04–96.52	89.05–94.53
*jak*							100	94.53–95.52	92.54–95.02	82.09	94.03–95.02	100
*lie*								98.51–100	94.53–98.51	83.08–84.08	96.52–98.51	94.53–95.52
*ochi*									94.53–100	81.59–84.58	94.03–98.01	92.54–95.02
*ram*										100	83.58–84.08	82.09
*vol*											98.51–100	94.03–95.02
This study												100

*arm, armillata; duk, dukei; fas, fassiata; fle, flexuosa; gib, gibsoni; gut, gutturosa; jak, jakutensis; lie, lienalis; och, ochegni; ram, ramachandrini; vol, volvulus.

**Table 2:** Sequence similarities in the mitochondrial 16S rRNA gene in the *Onchocera* sp. isolated in this study and other *Onchocherca* spp.*

Species	% Sequence similarity											
	*arm*	*duk*	*fas*	*fle*	*gib*	*gut*	*jak*	*lie*	*och*	*ram*	*vol*	This study
*arm*	100	91.56	96.00	96.00	94.67	93.78–94.22	94.22	93.33-93.78	93.33–93.78	93.33	91.55–92.00	93.78
*duki*		100	95.56	92.00	95.56	94.67–95.11	94.67	95.11	96.89–97.33	91.11	96.89–97.33	91.11
*fas*			100	95.56	96.89	96.00–96.44	96.44	96.44–96.88	95.56–96.00	93.33	94.66–95.11	96.00
*fle*				100	94.22	94.22–94.66	95.56	93.78–94.22	92.40–92.88	93.78	92.44–92.89	95.11
*gib*					100	97.78–98.22	97.78	96.89–97.33	94.67	93.78	93.77–94.22	97.33
*gut*						99.10–100	96.89–97.33	96.00–96.89	93.33–93.77	91.56–92.00	93.33–93.78	96.89–97.33
*jak*							100	98.22–98.67	92.88–93.33	92.44	92.00–92.44	99.56
*lie*								99.56–100	93.33–93.78	92.44–92.88	92.44–92.88	97.78–98.22
*och*									99.56–100	91.11–91.55	97.33–98.22	93.33–93.78
*ram*										100	90.22–90.67	92.00
*vol*											99.56–100	92.44–92.89
This study												100

*arm, armillata; duk, dukei; fas, fassiata; fle, flexuosa; gib, gibsoni; gut, gutturosa; jak, jakutensis; lie, lienalis; och, ochegni; ram, ramachandrini; vol, volvulus.

## Conclusions

The limiting factor in identifying the causative agent in our patient was the nature of the sample material. Because only a few formalin-fixed and paraffin-embedded sections were available, morphologic identification was not possible. PCR-based identification was restricted because DNA has a tendency to degrade when stored in formalin, which limits the length of the target sequence to ≈300 bp and limits its discriminatory power (*9*). A different approach with 3 PCRs, 1 for genus identification and 2 for species identification, and primers for highly variable multicopy targets enabled us to accurately identify the causative agent as *O*. *jakutensis*.

To our knowledge, *O*. *jakutensis* has never been identified as an agent of human filariasis. It has been identified as a rare parasite of red deer in Germany, Poland, and Russia, and may also be found in other northern European countries (*10*). Our patient came from the United States and had traveled all over Europe. She could thus have acquired the infection in several different locations.

Two findings for this patient were particularly unusual and obscured the identification of the parasite. The first finding was that she had, in contrast to all previous human cases of zoonotic onchocercosis, multiple nodules. The second finding was that her face (periorbital and buccal), neck, and scalp were affected, although zoonotic filariae are typically found in similar or identical tissues as in their natural hosts (*11*). *O*. *jakutensis* is usually found in tissues of the outer thigh and caudal part of the back; >2 nodules per infected host are rare (*12*,*13*).

It is unlikely that these findings are associated with greater virulence of *O*. *jakutensis* than of other zoonotic *Onchocerca* spp. However, parasite virulence might be related to the patient having had autoimmune disease since childhood and as a result having received long-term immunosuppressive therapy. The immune status of the patient was further impaired by renal insufficiency for >10 years. However, no data exist on the immune status of patients in any of the previously reported cases of infection with zoonotic *Onchocerca* spp. For other nematodes, e.g., *Strongyloides stercoralis*, a correlation between immune status of the patient and severity of disease is well established. One report describes more severe skin manifestations caused by *O*. *volvulus* in HIV patients (*14*).

We have identified a zoonotic infestation with an *Onchocerca* sp. that can cause disease in humans. The combination of impaired immunity and unusually progressing infestation highlights a new aspect of zoonotic filariasis.
